# “This thing of testing our blood is really very important”: a qualitative study of primary care laboratory services in Tete Province, Mozambique

**DOI:** 10.1186/s12939-016-0418-5

**Published:** 2016-08-24

**Authors:** Benvindo Toni Maria Tadeu, Diederike Geelhoed

**Affiliations:** 1 Provincial Health Directorate, Rua de Macondes, Cidade de Tete, Tete Province Mozambique; 2 Danish Ministry of Foreign Affairs, Provincial Directorate of Health, Rua de Macondes, Cidade de Tete, Tete Province Mozambique

**Keywords:** Clinical Laboratory, Mozambique, Universal Health Coverage

## Abstract

**Background:**

Laboratory services are essential for diagnosis and management of patients, and for disease control, and should form an integral part of primary health services capable of contributing to Universal Health Coverage. Nevertheless, they remain among the most neglected health services in resource-poor countries, including Mozambique. The Health Directorate of Tete Province, Mozambique, developed this study to analyse the role and perceived impact of laboratory services in primary healthcare on access, perceived service quality and disease control.

**Methods:**

Qualitative research was done in three primary health facilities with and three without a laboratory in Tete Province, purposively sampled for their available services, accessibility and size. The role of the laboratory in primary health care was explored by reviewing documents, including records and monthly reports, interviews with clinicians, laboratory technicians and key informants (community leaders), and through focus group discussions with beneficiaries. Numeric data were summarized in Microsoft™ Excel. Qualitative data were analysed for content within generated categories, interpreted within the concept of Universal Health Coverage, cross validated between the researchers.

**Results:**

The results showed a greater use of health services, with more frequent diagnosis and monitoring of prevalent diseases, in facilities with a laboratory as compared with facilities without. Clinicians, patients and community leaders in facilities with a laboratory perceived an improved possibility of diagnosing and treating prevalent diseases, resulting in greater satisfaction with the provided services. Laboratory technicians confirmed that patients appreciated having access to laboratory tests. Clinicians, patients and community leaders in facilities without a laboratory protested its lack, claiming that it increased the likelihood of costly referrals, delays and even deaths.

**Conclusions:**

The study concluded that the laboratory plays an important role in primary level health facilities, as it is associated with greater utilization and perceived higher quality of services. Lack of a laboratory hampers patient management, disease control and financial risk protection. Expansion of the clinical laboratory system at primary level health facilities should be a properly funded priority of the national health system in Mozambique and similar countries.

## Background

Adequate laboratory services support clinicians to provide quality medical care to patients, by performing tests to confirm diagnoses or to screen for diseases, facilitating optimization and effectiveness of treatment [1, 2]. Reliance on clinical diagnosis without laboratory confirmation lacks specificity and reliability, may lead to misdiagnosis in a considerable proportion of patients, and can even be associated with increased mortality [3]. Current international health regulations identify laboratory services as a core capacity of health systems, and it is recommended that all Member States of the World Health Organization (WHO) develop and maintain such services [4]. As such, laboratory services should form an integral part of primary health services capable of contributing to Universal Health Coverage (UHC), a concept incorporated in the recently approved Sustainable Development Goals (SDG) [2, 5].

In 2008 the WHO Regional Office for Africa supported the regional Consensus Meeting on Clinical Laboratory Testing Harmonization and Standardization in Maputo, Mozambique, resulting in the Maputo Declaration on laboratory systems, with the aim of addressing the laboratory challenges which limited the scale-up of services [1]. Since then, the need to expand and improve laboratory services has been recognised, within the larger context of health system strengthening in countries with limited resources. Limited laboratory capacity represented a major barrier to implementation and sustainability of health programs, including for HIV, malaria and tuberculosis [1, 6]. In response to the Declaration, some countries in Africa, such as Uganda, Rwanda, Nigeria and Tanzania, have elaborated national laboratory policies, defining the principal guidelines and priorities for the expansion and improvement of their laboratory networks, often combining the establishment of additional laboratories with effective and reliable systems for sample referrals [7–9].

Nevertheless, laboratory services remain a neglected component of the health system in Mozambique. A national laboratory policy has not been developed. The challenges include an insufficient number of trained laboratory staff, deficiencies in supply chains, inadequate infrastructures and a lack of quality management, related to inadequate allocation of government and/or donor funding to overcome these challenges [10]. As recently as 2013, only 23 % of health facilities in Mozambique had a clinical laboratory, which means that a large share of the predominantly rural population remains without adequate access to these essential services [10]. The limited laboratory capacity continues to hinder the effective implementation of health programs for HIV, malaria and tuberculosis, but also for neglected tropical diseases such as intestinal parasitosis and schistosomiasis.

Tete Province, located in central-western Mozambique, has approximately 2.500.000 inhabitants, with elevated levels of multidimensional poverty, while also presenting considerable inequalities between rural and urban areas. The province has a relatively under-resourced health system, with estimated deficits of around 40 % in human resources for health and 50 % in health facilities compared to national targets, higher than in most other provinces. Country statistics estimate the provincial HIV prevalence at 7 % (national prevalence: 11,5 %), while malaria, intestinal parasitosis and schistosomiasis are endemic in the province, as in the country. In 2014 the province had 116 health facilities, of which 36 % had a clinical laboratory [11]. Although the majority of the population live dispersed in rural areas (the provincial population density is just 25 per km^2^), about half of these laboratories are situated in facilities in provincial and district capitals, leaving more rural areas relatively underserved [11]. In Mozambique, public primary health care services are practically free at point of use, requiring only small financial contributions for care with exemptions for many vulnerable groups.

To support advocacy efforts for increased attention to the expansion of the primary care laboratory network in Mozambique, the Tete Provincial Health Directorate carried out this study to analyse the role of laboratory services in primary healthcare in relation to access, perceived service quality and disease control.

## Methods

### Study design and setting

A qualitative study was implemented to explore the role of the availability of laboratory services on access, perceived service quality and disease control in six rural primary healthcare facilities (HFs) in Tete Province (three with and without three a clinical laboratory). The facilities were purposively selected for their characteristics (availability of laboratory services, access from the provincial capital, size of target population, package of health services delivered and number of health personnel), aiming for general similarity apart from the existence of a clinical laboratory. As the selected HFs are situated in rural areas, while inequalities in multidimensional poverty are mainly between urban and rural areas rather than between different rural areas, health care needs were considered similar in the various target populations of the participating HFs. For the purpose of this study, the target population was considered to have access to laboratory services, when their HF included a functional and staffed clinical laboratory. A qualitative research design was chosen to explore the perspectives of services users and providers on the consequences of the availability, or lack thereof, of primary care laboratory services. The study was designed and implemented within on-going efforts to build local capacity in scientific research, and it presented a first experience in the use of qualitative methods for one of the principal investigators and both research assistants. The design and implementation of the study respected all recommended methodological procedures in the literature.

### Data collection

Data collection consisted of a structured document review of routine monthly reports and record books from the HFs and their laboratories by the principal investigator, assessing the volume of services provided and diagnosed diseases, from January to June 2014. In addition, we conducted 15 semi-structured individual interviews with laboratory technicians, clinicians and community leaders, and six focus group discussions with patients. Each focus group discussion had between four and six participants and lasted approximately one and a half hour. Volunteer participants were invited from among the patients attending the visited health facility, excluding any acutely sick patients. All laboratory technicians and clinicians attending outpatient consultations at the participating HFs were invited for an interview. The community leaders invited for interview were those known at the HFs as representatives of the local communities for matters involving the health services. The interviews and discussions, held in Portuguese or local language as appropriate, were facilitated by two previously trained research assistants, familiar with local health services, but not having any hierarchical relation with the participant health personnel. One assistant guided the conversations, while the other took notes and attended the recordings. All conversations were held in or close to the health facility, in a private setting, at the end of the work day, taking care not to interfere with the usual attendance to patients. The semi-structured interview and discussion guides included questions soliciting views about the perceived usefulness and importance of the laboratory services, the utilization of its services, perceived difficulties for its use, and suggestions for change or improvements. In health facilities without a laboratory, the questions also asked for opinions on how the lack of these services affected the healthcare provided, and how useful alternatives such as sample referral systems were perceived. All interviews and discussions were recorded and subsequently transcribed in Portuguese by the facilitators. Data were collected until saturation was reached.

### Data analysis

The numeric data from reports and record books, excluding patient identifying information, were analysed in Microsoft™ Excel. The mean number of consultations, diagnoses and laboratory tests per month, as well as their rate per 1.000 target population, were calculated for the different HFs and overall for both study groups. These means and rates were then arranged in tables and examined for similarities and differences between HFs with and without a laboratory. We also estimated their implications for current utilization of laboratory capacity and for disease control in the target populations. The data collected from interviews and focus group discussions were manually analysed for content, generating categories of recurring ideas and phrases expressed by the participants. The three main categories generated within this content consisted in the utilization of laboratory capacity and perceived quality of diagnosis and treatment among health professionals; perceived quality of care among patients and community leaders, specifically, perceived effectiveness and timeliness of care; and the financial implications of access to laboratory services or the lack thereof. Subsequently, we examined the text fragments gathered within each of these categories for underlying meanings, which were interpreted within the concept of UHC as stated in the first part of target 3.8 of the SDG: “Achieve universal health coverage, including financial risk protection, access to quality essential healthcare services (and access to safe, effective, quality and affordable essential medicines and vaccines) for all” [5]. The final interpretations were reached by cross-validation between the understandings reached from the different data sources, based on consensus between the two researchers, taking the viewpoint of the research assistants into account.

## Results

The two study groups of three HFs each included in our study, in six different districts and outside provincial and district capitals, had similar characteristics in terms of average target population and number of health personnel, package of services, and access (Table 1). In the six HFs, routine data collection tools, record books and monthly reports of the months January to June 2014 were reviewed, including outpatient records, monthly reports of HIV and tuberculosis services, monthly reports of maternity services, laboratory registration books. A total of 53 participants were interviewed or collaborated in the focus group discussions (Table 2). From the content of the semi-structured interviews and focus group discussions, transcription, lecture and coding, and analysis yielded the information on the perception of service quality among health professionals and service users, including timeliness and perceived effectiveness, and on the financial implications of access to services for the latter group.

**Table 1:** Characteristics of participating primary HFs

	Mean target population	Access and mean distance from provincial capital	Mean number and range of health personnel	Service package	Laboratory capacity
HFs with a laboratory (A, B, C)	35.577 range: 7.066 – 52.109	232 km, partly by dirt road	6 range: 4–9	All HFs: outpatient care, maternity, HIV and tuberculosis treatment, pharmacy; one with inpatient services;	All HFs: • Haemoglobin • manual leucocytes count in peripheral blood smear • Rapid Plasma Reagin • Ziehl-Nielson • thick blood smear • microscopy for urine and faeces parasitology • rapid tests for HIV, malaria • T-cells Cluster of Differentiation 4 (Alere Pima™, resident in one HF and regular outreach visits in the two others) • Polymerase Chain Reaction of HIV DNA sample collection for referral One HF with blood transfusion service
HFs without a laboratory (D, E, F)	34.222 range: 8.348 – 51.380	155 km, partly by dirt road	5 range: 4–6	All HFs: outpatient care, maternity, tuberculosis treatment; two with HIV treatment; one with pharmacy	All HFs: • rapid tests for HIV, malaria • T-cells Cluster of Differentiation 4 (Alere Pima™, regular outreach visits) • Polymerase Chain Reaction of HIV DNA sample collection for referral One HF with haemoglobin test

**Table 2:** Distribution of participants in interviews and focus group discussions

	Number of participants		
	Community leaders	Patients	Health personnel
HFs with a laboratory (A, B, C)	4	17	7
HFs without a laboratory (D, E, F)	6	14	5
Total	10	31	12

### Service utilization and disease control

The reviewed documents indicated a higher level of health service utilization in the HFs with a laboratory, with nearly twice as many consultations and childbirths attended, four times more patients starting antiretroviral treatment (ART), and three times as many patients starting treatment for tuberculosis, per 1.000 target population per month, as compared to HFs without a laboratory (Table 3).

**Table 3:** Service utilization in both groups of HFs

	Mean number per month (mean number per month/1.000 target population)			
	Consultations	Childbirths	Patients starting antiretroviral treatment	Patients starting tuberculosis treatment
Three HFs with a laboratory (A, B, C)	6.816 (63,9)	152 (1,4)	182 (1,7)	12 (0,1)
Three HFs without a laboratory (D, E, F)	3.594 (35,0)	87 (0,8)	41 (0,4)	4 (0,03)

We also observed important differences in the pattern of diagnosed diseases between the two types of HFs. Taking the different availability of the various tests into account, HFs with a laboratory performed more tests for malaria (36,9 vs. 15,3 per 1.000 population per month), and also tested more people for HIV (both with rapid tests and Polymerase Chain Reaction DNA; 6,1 and 0,1 vs. 3,7 and 0,02 per 1.000 population per month), with a higher positivity rate (7,9 % vs. 2,5 %), than HFs without a laboratory. They also performed more tests to assess immune response in patients in ART (3,3 vs. 0,1 per 1.000 population per month) and to check for anaemia (2,9 vs. 0,1 per 1.000 target population per month). In addition, HFs with a laboratory had the capacity to confirm the diagnosis of a considerable number of patients with urinary and intestinal parasitic infections (0,6 and 0,2 per 1.000 population per month, respectively), which was not possible in the HFs without a laboratory. Sputum examinations for tuberculosis resulted in more patients identified with this disease in the HFs with a laboratory than in those without a laboratory, which depended on sample referral (5 vs. 1 patients per month, Table 4).

**Table 4:** Tests performed and diseases diagnosed in both groups of HFs

	Malaria				Haemoglobin: Mean number tested per month (mean number per month/1.000 target population)	HIV				Parasitosis		Tuberculosis	
	Thick blood smear		Rapid test			Rapid test		CD4: Mean number tested per month (mean number per month/1.000 target population)	PCR HIV DNA: Mean number tested per month (mean number per month/1.000 target population)	Intestinal: Mean number tested per month(mean number per month/1.000 target population)	Urinary: Mean number tested per month(mean number per month/1.000 target population)	Mean number tested per month (mean number per month/1.000 target population)	Mean number positive per month (%)
	Mean number tested per month (mean number per month/1.000 target population)	Mean number positive per month (%)	Mean number tested per month (mean number per month/1.000 target population)	Mean number positive per month (%)		Mean number tested per month (mean number per month/1.000 target population)	Mean number positive monthly (%)						
Three HFs with a laboratory (A, B, C)	132 (1,2)	44 (33,2 %)	3.935 (36,9)	2.262 (57,5 %)	304 (2,9)	649 (6,1)	51 (7,9 %)	354 (3,3)	9 (0,1)	21 (0,2)	68 (0,6)	37 (0,3)	5 (14,0 %)
Three HFs without a laboratory (D, E, F)	N/A	N/A	1.566 (15,3)	977 (62,4 %)	13 (0,1)	375 (3,7)	10 (2,5 %)	10 (0,1)	2 (0,02)	N/A	N/A	4 (0,04)^a^	1 (23,1 %)^a^

N/A Not Available

^a^ via sample transfer to neighbouring HFs with a laboratory

The number of tests performed each month in the three HFs with a laboratory was nearly three times as high as that performed in the three HFs without a laboratory, not only because of the additional testing capacity in the laboratory itself, but also because more rapid tests for HIV and malaria were performed by clinical staff during consultations. As the number of consultations per month was only about twice as high in the HFs with a laboratory than in those without, part of this difference might be explained by improved clinical practices, with less reliance on clinical diagnosis without laboratory confirmation. It was not clear whether the presence of a laboratory technician also improved the quality of testing with rapid tests by clinicians, as the positivity rate for malaria rapid tests was rather similar between the two groups of HFs, while the higher observed positivity rate of HIV rapid tests might also be related to possible differences in HIV prevalence in the target populations.

The utilization of the available laboratory services was moderate, as the presence of laboratories in the three HFs added approximately 3.000 tests per month compared to the HFs without a laboratory, which corresponded to approximately 50 tests performed per working day per laboratory. As a large proportion of these tests were rapid tests for HIV or malaria, partly performed by clinicians, spare capacity might still exist for the additional performance of more time-consuming examinations, such as thick blood smears for malaria or tuberculosis, microscopy for urine and faeces parasitology, or manual leucocytes count in peripheral blood smears.

From the perspective of disease control, the difference between the two groups of HFs in the number of positive malaria tests indicated that, per month, over 1.300 people more were diagnosed with malaria with laboratory confirmation by the three HFs with a laboratory than by the three HFs without a laboratory, despite their very similar target populations and expected malaria incidence. The study collected data which included the peak malaria season during the months of January to March, which is the period of rains locally. Although some people attended in the HFs without a laboratory might have been treated for malaria based on clinical suspicion only, the data suggest that, every month, possibly hundreds of people suffering from malaria might have gone undiagnosed and untreated among the target population of the HFs without a laboratory. Similarly, albeit affecting smaller numbers of people, every month tens of patients might have gone untreated for HIV or tuberculosis in the target population of the HFs without a laboratory. The presence of such number of people with untreated malaria, HIV or tuberculosis in this target population, likely limited the effect of local disease control efforts during the same period (such as bed net distribution, in-door spraying, condom distribution, behaviour change communications).

### Perceived service quality

#### Health professionals

The clinicians generally thought that the availability of a clinical laboratory enabled them to deliver services with improved professional quality, particularly considering diagnosis and treatment of the most common diseases. They felt that the laboratory results facilitated their decision making regarding optimal clinical management. In addition, they pointed out that the availability of a laboratory permitted a more expedient attendance. With laboratory services present, diagnoses could be made more quickly and treatment started without delays, as there was no lengthy process of referral required for samples or patients to any other HFs for laboratory services. The perception that they were able to deliver quality care to their patients, leading to recognition by their target population, was a source of professional satisfaction, while nobody mentioned or complained that such increased valorisation by the population of their services might be associated with an increased workload.

***Clinician (HF A-with laboratory)****: “The clinical laboratory is very important in this health centre, because it improves the quality of our work, it facilitates the diagnosis of many diseases and the decision to treat them safely. This allows for quicker attendance and it avoids many transfers of patients”.*

***Clinician (HF E-without laboratory)****: “We have many limitations in the diagnosis of many diseases here, and this determines the treatment and service we provide to our patients. For example, we do not offer tuberculosis or HIV treatment, because of the lack of a clinical laboratory. We have to transfer patients often to* [another health facility] *that does have a laboratory.”*

Clinicians in the HFs with a laboratory expressed satisfaction with the available tests and called even for an increase in capacity, to include more tests and services (for example, biochemistry and transfusion capacity), to further reduce the need for sample referral and patient transfers. The laboratory technicians did, however, recognize that some tests were underused, such as microscopy for parasites in faeces and urine, or manual leucocytes counts in peripheral blood smears. Furthermore, they lamented about difficulties in the logistic arrangements for sample referrals, and demanded improvements in supply chain management to avoid interruption of activities due to stock-outs. Health personnel in HFs without a laboratory complained about the limitations experienced in diagnosis, treatment and monitoring of patients, and called for the urgent installation of laboratory services in their HF. Both groups of clinicians called for more functional blood transfusion services, and stated that they often needed to transfer women with complications of childbirth to other, usually distant, HFs with more readily available blood transfusion services. Though rare, they confirmed that some maternal deaths had occurred as a result from the difficulties to arrange timely transfusions.

***Clinician (HF B-with laboratory)****: “We want to do blood transfusions here, biochemistry, CD4, and we should also improve the supply of reagents, because we have many stock-outs.”*

***Clinician (HF D-without laboratory)****: “They should allocate a clinical laboratory in this HF, because you see, there are many activities that we do not perform due to a lack of this service. It would make such a difference, because it would surely improve our performance in diagnosis, treatment and management of diseases.”*

In summary, in the perspective of health professionals, especially the clinicians, service quality benefitted considerably from the availability of a clinical laboratory, permitting to better diagnosis and management of patients, and more expedient attendance, despite limitations in the laboratory’s utilization and performance. The absence of a laboratory presented a source of frustration for health professionals, as they could not perform to their full knowledge and capacity to alleviate the suffering in their patients.

#### Service users

Patients and community leaders claimed that they had more confidence in the diagnosis and treatment of the health facilities, when these included laboratory tests. Such tests permitted them to obtain reliable information regarding the nature of their illness, rather than having to depend solely upon the explanations of the attending clinician, however educated that clinician might be. They believed that they were more likely to receive the correct medication for their illness after being tested at the laboratory, and felt more convinced of its effectiveness and ability to cure the illness in question. As so many patients live far from a HF, this trusted effectivity of prescribed medications was considered important, to ensure a speedy recuperation, and avoid having to come back after potential treatment failures.

***Patient (HF C-with laboratory):****“This thing of testing our blood is really very important.* […] *We like to have blood tests in the laboratory, when we come here in the hospital. Oh yes, we are sure that they will check our blood to know what* [disease] *we really have and then give us the correct medications.”*

***Community Leader (HF A-with laboratory):****“Before the laboratory was here, the nurses gave us medications only based on the explanations from the patients, the same as traditional healers and diviners, but now we receive medications based on the result of the analysis”.*

***Patient (HF E-without laboratory):****“A place to perform* [laboratory] *tests is very important, because when a person is ill, he does not really know what is causing the problem, and although the doctor may be well educated and might say that it is because of this or that, that’s only talking, it is necessary to do tests to know the truth of what is happening, to check whether the strength or the velocity of my blood is good or not. That is why it is important for a Health Facility to have a place to perform* [laboratory] *tests.”*

***Patient (HF F-without laboratory):****“There is no laboratory here, and patients are given medication according to explanations* [of the clinicians]*, they are like traditional healers! Sometimes it is different from the illness the person really has, and then he has to come back another time; we live far from here, and then we have to leave our farms to come back many times with the same illness.”*

In addition, patients and community leaders from HFs without a laboratory expressed dissatisfaction with the delays in their diagnosis and treatment related to the need for referral of samples or travel to another HF for basic laboratory tests. Such delays were not just considered inconvenient, but even dangerous, as the illness might progress considerably before any correct treatment might be received. Remarkably, none of the participants, neither service providers nor service users, referred to the use of point-of-care tests to overcome part of the limitations associated to the lack of laboratory services, although these tests were available for the diagnosis of malaria and HIV.

***Patient (HF D-without laboratory):****“*[If there were a laboratory here]*, then we would be tested here, and there would be no delays in receiving treatment, because we would receive our test results that same visit, and we might be attended at the same time. Now, without a laboratory, a person might end up dying without knowing his test results and without having taken medication, because the test results* [of samples transferred elsewhere] *delay a lot, and that is very difficult.”*

From these contributions it was clear, that the patients in these rural areas considered that a clinical laboratory formed an essential part of quality modern biomedical health services, an important added value compared with the locally available traditional medicine. Patients clearly trusted that, with laboratory examinations, their diagnosis and treatment would be correct and expedient, minimizing time spent waiting or travelling for health care, and leading to a quick recovery. Without laboratory examinations, however, the value attributed to the health care received would not exceed that of traditional medicine, and as such, offer possibly less compensation for the long waiting and travelling times.

### Financial implications of access to services for patients

Patients and community leaders from HFs without a laboratory reported that they were facing considerable financial hardship related to the lack of a laboratory, including blood transfusion services. Even clinicians referred to the fact that they encountered regularly patients who were unable to afford the costs of traveling to a neighbouring HF when a laboratory was not available locally. Many participants mentioned the need to have to sell farm produce to be able to gather the necessary funds for travel to a HF with laboratory services.

***Community Leader (HF A-with laboratory):****“We are very pleased with* [the installation of a local laboratory]*, because before we needed to sell our agricultural produce, such as corn or beans, to be able to travel in search of a laboratory”.*

***Patient (HF E-without laboratory):****“There is no place to test our blood here, so sometimes we are sent to* [another HF] *to perform tests, and that is not good, because sometimes we have no money for transport, and then we have to sell corn or beans or potatoes.”*

***Patient (HF D-without laboratory):****“We can only have tests in* [the district capital]*, and we’ll have to sell chickens or goats to manage to reach the laboratory. Otherwise, we’ll just die.”*

Although the laboratory services in themselves might be free at point of use, access was clearly carrying considerable financial implications for patients required to travel to another HF for laboratory services, due to the required transport costs. The participants recognized that these costs could not always be assumed by everybody, sometimes with dire consequences. Obviously, the poorest in these generally underprivileged communities would be at highest risk of not being able to afford the necessary travel costs. The availability of laboratory services at each and all rural HFs would therefore contribute considerably to the financial risk protection required for Universal Health Coverage.

## Discussion

The study results show that, in HFs with a laboratory, there was greater utilization of health services, with more and improved diagnosis, treatment and follow-up of common diseases, compared to HF without a laboratory, despite an otherwise very similar service package for similarly sized rural target populations, with probably similar health care needs. The numbers of patients affected by the lack of laboratory services at their local HFs were considerable, depending upon the type of disease. In particular, malaria would not be diagnosed with laboratory confirmation in more than 1.000 patients per month in the target populations of the three HFs without laboratory, while also tens of patients with HIV or tuberculosis might have had to forego diagnosis and treatment every month in these same areas. It is unlikely that the target populations using a HF without a laboratory would be so much healthier than those using a HF with a laboratory. The expected differences in incidence and prevalence of diseases like malaria, HIV, tuberculosis, anaemia, parasitic infections, or complicated childbirth would be small between these similar and predominantly rural areas in the same province, although some transfer of neighbouring populations without access to laboratory services probably occurs. The most likely interpretation, therefore, would be that, in HFs without a laboratory, diseases go often undiagnosed and remain without treatment, possibly contributing to increased morbidity, mortality, and reduced disease control in the populations without laboratory access. These results confirm that laboratory diagnostic capacities, as a key component of health systems, are essential for effective surveillance of diseases, as well as for their treatment [1, 3, 4, 12].

Remarkably, in HFs with a laboratory more tests were done per 1.000 target population, even in the case of diseases which do have rapid point-of-care tests (POC) in use in Mozambique. The study findings suggest that the use of rapid tests for malaria, HIV and anaemia in HF without a laboratory, do not adequately replace the performance of a clinical laboratory. For clinicians it might be difficult to achieve the same testing quantity and quality with rapid tests, due to their different time allocation and priorities compared to laboratory personnel. In the high volume and understaffed health facilities of this study, rapid tests are often not rapid enough, and a separate service with the exclusive duty of performing tests might therefore yield more accurate results. Other studies have already shown that despite clear advantages, POC testing has important limitations, and laboratory-based testing is likely to continue to be an important component of future diagnostic networks [13, 14].

Another important result of this study is that patients had more confidence in health care delivered in HFs with a laboratory, while patients attended in HFs without a laboratory protested its lack of availability, claiming that it results in inadequate care or difficult and expensive journeys due to referrals, and even in deaths. The higher value attributed to health care with laboratory tests is likely to form an important explanation for the greater utilization of the HFs with a laboratory observed in this study. In addition, clinicians in HFs without a laboratory felt underutilized and unable to perform their tasks to the professional standards for which they were trained, which surely would be a contributing factor to prevailing low job satisfaction and motivation, added to other known factors, such as low salaries, poor staff housing and inadequate supervision support [15].

Viewed within the proposed target for Universal Health Coverage of SDG 3 (Ensure healthy lives and promote well-being for all at all ages), these findings suggest that it might be difficult to achieve target 3.8 of the SDG: “Achieve universal health coverage, including financial risk protection, access to quality essential healthcare services […] for all” [5], without universally providing laboratory services at primary care level, also in rural areas. It becomes clear that the allocation of laboratories cannot be seen as a luxury reserved for urban hospital settings, but must be executed throughout the entire national health system, including in rural primary health care facilities.

To be able to move forward towards SDG target 3.8 in Mozambique, an expansion of appropriate laboratory infrastructure would be necessary, consisting of space where health professionals can perform analytical tests, to be defined and allocated on the basis of number and types of analytical tests to be performed within the HF [16]. Functional electricity and water supplies should be reliably available to allow uninterrupted modern testing services within each HF, including those of primary care level. In addition, the investment in appropriate instrumentation, automated laboratory equipment, and reliable consumable’s supply chains is crucial for sustainable laboratories [17]. To achieve such conditions in poor-resourced countries, such as Mozambique, would require substantial and continuous funding of a well-designed National Laboratory Policy. This policy would also have to consider the training and placement of corresponding numbers of laboratory technicians of appropriate technical capacities, to ensure that the clinical laboratories built and equipped would be adequately staffed, in combination with other prerequisites, including improved supply chain management, supervision and technical support, improved quality assurance and information systems, in order to ensure a viable decentralised laboratory system [18, 19].

Limitations of this study include the fact that the principal investigators originated within the public healthcare system in the province, which likely coloured the responses of both service providers and users, although efforts were made to avoid hierarchical relations, such as the use of research assistants not in hierarchical relationship with the participating HF staff. In addition, disease statistics from populations in areas without laboratory might have spilled over to HFs with a laboratory, as for sicker patients it would be more imperative to travel and look for services elsewhere. Their test results might thus inflate the utilization and positivity rates in HFs with a laboratory. A study design with ‘before and after’ analysis might have been more useful to illustrate the impact of laboratory services. However, due to the slow pace of laboratory service expansion, the many other interventions routinely done to affect service quality and utilization, and the frequent changes in the health information systems used in Mozambique, which hinders comparison of data over time, such design was judged unfeasible.

As this is a qualitative study, in the specific situation of Tete province in Mozambique, caution is warranted in the generalisation of these results to the entire country or African region. Still, the study provides information which is consistent with existing literature on the topic. The setting of the six participating HFs is typical of many others in rural areas with low population density in Mozambique and in other low-income countries in the region, where health care resources, including infrastructures, health care professionals, and more complete service packages, tend to be relatively concentrated in more urbanized areas, and where long travel distances limit access to services for rural populations [20, 21]. It forms a case study which illustrates, again, that the recommendations of the Maputo Declaration in 2008 are still valid, and need to be transformed into action in Mozambique and other similar low-income countries.

## Conclusions

This study shows that laboratory services are considered very important for access, health care quality and disease control in rural populations in Tete province, Mozambique. Therefore, the expansion of the laboratory network should be an appropriately funded priority of the national health system. A National Laboratory Policy in Mozambique would be able to establish rules for the expansion of the laboratory network and calculate required costs to facilitate the mobilization of funding. Parallel to the laboratory network expansion, enhanced supply chain management is essential to ensure the provision of uninterrupted services in established laboratories, ensuring maximum utilization and quality of the currently low installed capacity.

These recommendations require a serious investment in the area, so key stakeholders have an important role to play: national health authorities must set policies and priorities, while government and development cooperation partners should allocate the required resources. Only then the laboratory services will be able to contribute fully to achieving Universal Health Coverage as a core capacity of the national health system [1, 3, 4].

## Abbreviations

ART, Anti-Retroviral Treatment; HF, Health Facility; HFs, Health Facilities; HIV, Human Immunodeficiency Virus; SDG, Sustainable Development Goals; UHC, Universal Health Coverage; WHO, World Health Organization.
